# Development of an optimal relief method for the palatal plate by stress analysis

**DOI:** 10.1186/s12903-021-02014-z

**Published:** 2021-12-20

**Authors:** Tomoko Mukai, Yuji Sato, Osamu Shimodaira, Junichi Furuya, Akio Isobe, Tomoka Omori

**Affiliations:** Department of Geriatric Dentistry, School of Dentistry, Showa University, Ota, Tokyo, 145-8515 Japan

**Keywords:** Palatal mucosa, Relief, Simulation, Three-dimensional finite element analysis, Stress distribution, Borderline

## Abstract

**Background:**

Plate dentures cannot be easily modified after fabrication; therefore, the sites and magnitude of relief must be effectively assessed at the time of fabrication. However, a considerable variation exists in the magnitude of optimal relief and relief range, and there are no guidelines that present these clearly, leading the dentists to decide subjectively. Thus, this study aims to develop an optimal relief method to improve the stress bearing capacity of the palatal mucosa.

**Methods:**

The objective of this study, namely, the borderline, was set in steps. A three-dimensional finite element model for the pseudopalatal plate was created and used to evaluate the changes in stress distribution in the palatal mucosa due to the selective relief of stresses above the borderline. The resulting data were used to develop the optimal relief method.

**Results:**

In the relief model with a borderline of 0.04 MPa or higher, the distribution volume at which a high stress of 0.20 MPa or higher is generated was approximately 800% of that with the no-relief model, and in the relief model with a borderline of 0.06 MPa or higher, the respective ratio was approximately 280%. On the other hand, the relief models with a borderline of 0.14 MPa or higher were approximately 60%. In the mid-palatal relief model, the distribution volume at which a stress of 0.20 MPa or higher was generated was 180% of that in the relief model.

**Conclusions:**

The supportive strength of plates can be increased by selectively applying optimal relief rather than standard relief, allowing for easier and more effective plate-denture treatment.

## Background

With the aging population in Japan, the demand for removable denture prosthetics is increasing [1]. High-quality plate-denture treatment is linked to an increased quality of life for patients. For plate-denture treatment, an objective evaluation of the properties of the denture-supporting mucosa is important.

Recently, plate dentures with frameworks of cobalt–chromium alloy and zirconia have been widely used for their increased durability and comfort while in use [2, 3]. However, after the fabrication of these dentures, it is difficult to modify them by adjusting; therefore, it is essential to sufficiently assess the sites and magnitude of relief at the time of fabrication. In practice, in connection with complete denture treatment in the past, as a part of denture design, relief is provided in the mid-palatal area. This relief is an important means of preventing pain due to the denture plate in the region of mucosal thinning, ensuring denture stability, and preventing denture damage, as well as preventing compression injury to the nerves and blood vessels [4, 5]. However, a considerable variation exists in the magnitude of optimal relief and relief range, and no guidelines are available that present these clearly, leading the dentists to decide subjectively.

To optimize denture design for individual patients, a system has been developed by fitting a strain gauge to an ultrasonic thickness gauge so that changes in load and mucosal thickness causing pain are measured simultaneously [6]. Further, the relationships between the properties of the denture-supporting mucosa (thickness and elasticity) and the pain threshold (pressure, degree of subsidence, and compression rate) in dentate and edentulous persons have been analyzed [7, 8]. In addition, to apply this system clinically, a maxillary-palate-shaped device has been developed for the simultaneous measurement of bite force and palatal mucosal subsidence at the time of pain onset in dentate persons. By using this device, the relationships between bite force and palatal mucosal subsidence in dentate persons were analyzed simultaneously, and the effects of palatal relief on palatal-mucosal-supporting strength were clarified [9]. Palatal mucosal stress analysis in a simulation based on three-dimensional (3D) finite element analysis (FEA) is effective for the objective evaluation of various types of denture-supporting mucosa [10].

In this study, the borderline was defined as the cutoff value of the stress generated in the palatal mucosa for relief; the borderline was set in steps in this study. The changes in stress distribution in the palatal mucosa due to the selective relief of stresses above the borderline were evaluated via a 3D finite element simulation. The purpose of this study was to develop an optimal relief method to improve the stress bearing capacity of the palatal mucosa.

## Methods

### Subject and region of interest

The subject was a dentate person with no abnormality in the palatal mucosa or presence of a marked tori. This person has participated in previous studies in which mucosal elasticity and thickness were measured. Further, a 3D finite element model was prepared. Similar to previous studies, the region of interest was defined as the palatal mucosa extending on the maxilla from a position mesial to the first molars on the left and right side to a position distal to the second molars on the left and right [9, 10]. This study was conducted in accordance with the principles of the Declaration of Helsinki. The informed consent form was approved by the Ethics Committee of Showa University (approval number 2014-036).

### Establishment of 3D finite element models

**(1) Establishment of the pseudopalatal plate model**

**Fig. 1:**
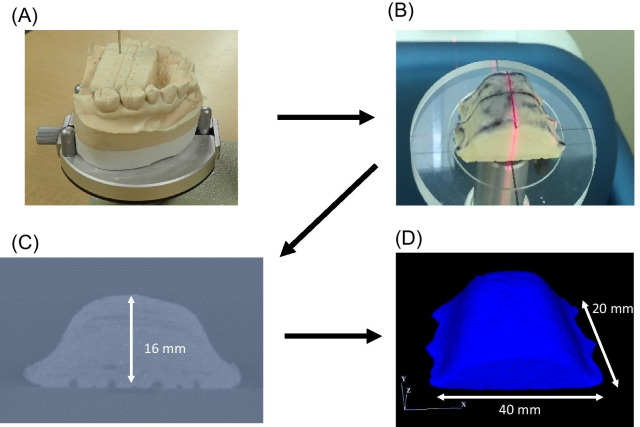
Constructing the three-dimensional finite element model. **A** Pseudopalatal plate using scanning resin; **B** CT imaging; **C** captured by 3D-FEA software; **D** three-dimensional finite element model of the pseudopalatal plate

The pseudopalatal plate of the device for the simultaneous measurement of the palatal region was prepared using X-radiographically observable scanning resin, and computed tomography was performed. A 3D finite element model for the pseudopalatal plate was created from the computed tomography data obtained using a 3D FEA software (Mechanical Finder®; Research Center of Computational Mechanics, Inc., Tokyo, Japan; Fig. 1). This was referred to as the no-relief model [10].

**(2) Relief settings**

(1) Setting the borderline for the stress

**Fig. 2:**
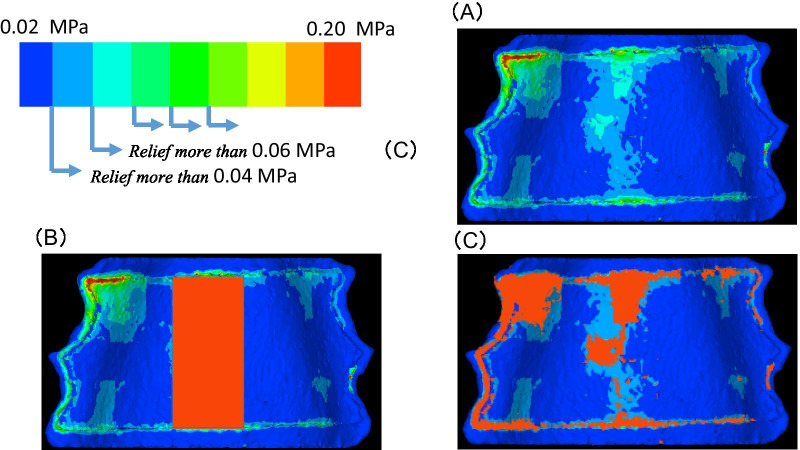
Relief setting. **A** No relief; **B** mid-palate relief; **C** relief more than 0.06 MPa

In a previous study, the pain generating bite force was measured as 111 N [10]. This force was applied to the center of the no-relief model, and the value of stress thus generated in the palatal mucosal model at that time, 0.02 MPa, was set as one end of the range for bite force in the simulation, with 0.20 MPa as the other end. In other words, the bite forces were set as 0.04, 0.06, 0.08, 0.10, and 0.14 MPa. The stress generation ranges at each bite force or higher were considered to be the target ranges for relief (Fig. 2).

(2) Relief in ranges within which stress is generated at borderline or higher

Based on the range in the no-relief model where stresses above the borderline were generated, this model was trimmed. Then, a relief of 0.25 mm [11] was applied to this trimmed area to create the outline of the pseudopalatal floor model with relief. Each of the resulting model was categorized as the 0.04 MPa or higher, 0.06 MPa or higher, 0.08 MPa or higher, 0.10 MPa or higher, and 0.14 MPa or higher relief model (Fig. 2).

(3) Single relief for the general mid-palatal area

Based on the no-relief model, relief was applied to breadths of 10 and 0.25 mm in the maxillary mid-palatal area, thereby providing a single relief to the maxillary mid-palatal area of the external form of the pseudopalatal plate model. This is referred to as the mid-palatal relief model (Fig. 2).

### Setting the external form of the palatal mucosa model

The mucosal surface in the pseudopalatal plate model was divided into 14 segments consistent with the measurement sites, and the measured palatal mucosal thickness was added as an element. In addition, the segments were edited to ensure smoothness [10].

### Mesh formation

To ensure consistency with previous studies [10], tetrahedral meshing with a size of 0.5 mm was chosen in this study. The mesh was created using the automatic creation function of the Mechanical Finder® software with 88,951 nodes and 475,962 elements in total. In addition, both the pseudopalatal plate model and the palatal mucosa model were considered to be homogeneous and isotropic linear elastic structures.

### Analysis conditions

(1) Setting physical values

The physical properties of the resin in a humid environment were evaluated using a pseudopalatal plate model. The elasticity was assumed to be 2650 MPa [12], and the Poisson's ratio was 0.3 [13]. The elasticity calculated from the measurements of the subject was applied to the palatal mucosa model [10].

(2) Loading conditions

**Fig. 3:**
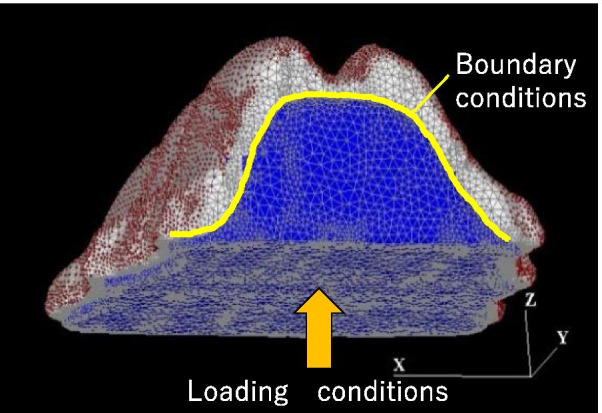
Loading conditions and Boundary conditions

The loading was vertical onto the center of the pseudopalatal plate. With respect to the load, when the subject developed pain, the bite force was 111 N [10] (Fig. 3).

(3) Restraint conditions

The uppermost surface of the palatal mucosa model was assumed to adhere to the maxillary bone, and the model was fully constrained [10].

(4) Boundary conditions

The boundary conditions in the pseudopalatal plate model and palatal mucosa model were considered as the adhesion conditions [10] (Fig. 3).

### Analysis target

In the palatal mucosa model, the volume of the range within which von Mises stress was generated at each borderline or higher was calculated, and the results were compared between each condition, with and without relief.

## Results

### Comparison of von Mises stress generated in relief and no-relief palatal mucosa models

**Fig. 4:**
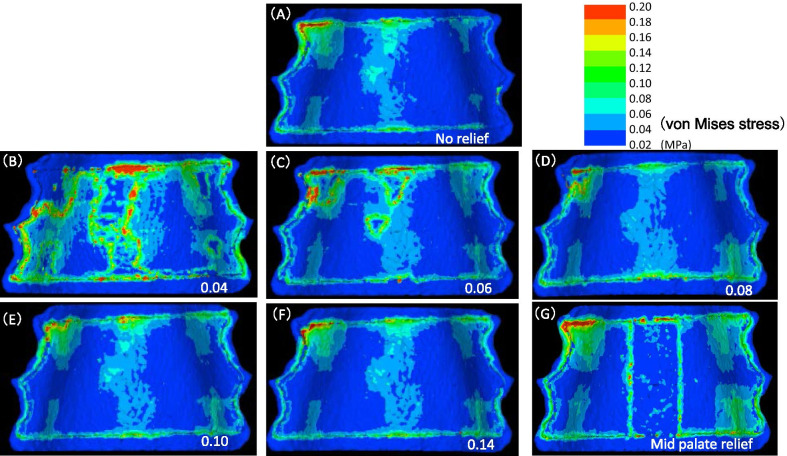
Distribution map of von Mises stress. **A** No relief; **B** relief more than 0.04 MPa; **C** relief more than 0.06 MPa; **D** relief more than 0.08 MPa; **E** relief more than 0.10 MPa; **F** relief more than 0.14 MPa; **G** mid-palate relief

The distributions of von Mises stress generated in the relief and no-relief palatal mucosa models are shown in Fig. 4.

In this study, von Mises stress was used instead of principal stress because the objective was to know the stress distributed throughout the palatal mucosa.

In the relief model with borderline at 0.04 MPa or higher and 0.06 MPa or higher, the stress distribution was higher toward the front of the mid-palatal area than in the no-relief model. In the relief model with borderline at 0.08 or higher, 0.10 or higher, and 0.14 MPa or higher, no marked changes were found in comparison with the no-relief model. In addition, in the mid-palatal relief model, stress distribution was at the margin of the relief range and at both margins of the palatal plate.

### Distribution volume ratios for each stress values

**Fig. 5:**
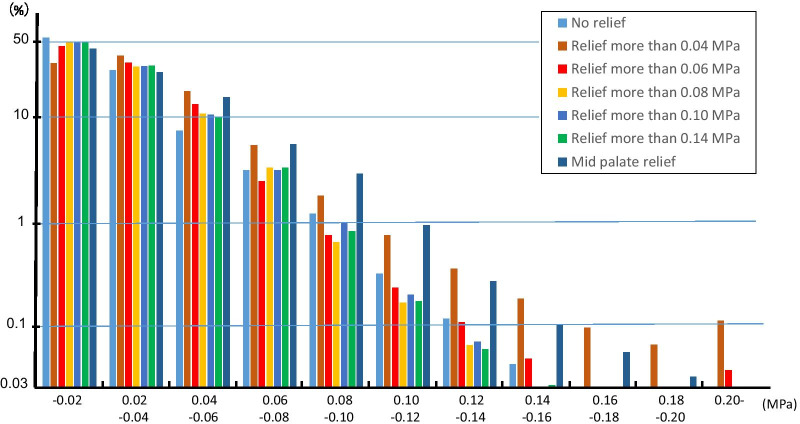
Volume ratio of stress distribution at each stress value

The distribution volume ratios for each stress value on the palatal mucosa are shown in Fig. 5. With a borderline of 0.04 MPa or higher and 0.06 MPa or higher, the relief model was found to increase the distribution volume of stress at 0.20 MPa or higher. In the relief model with a borderline of 0.08 MPa or higher and 0.10 MPa or higher, the stress distribution was at 0.14 MPa or lower. In the relief model with a borderline of 0.14 MPa or higher, the stress distribution at 0.08 MPa or higher decreased, and stress at 0.06 MPa or lower increased. In the mid-palatal relief model, the distribution volume of stress at 0.04 MPa or higher increased in comparison with that in the no-relief model. In addition, a comparison of the relief models with each borderline showed maximum increases in stress distribution volume between 0.06 and 0.12 MPa.

### Distribution volume ratios for each stress value in the relief model

**Fig. 6:**
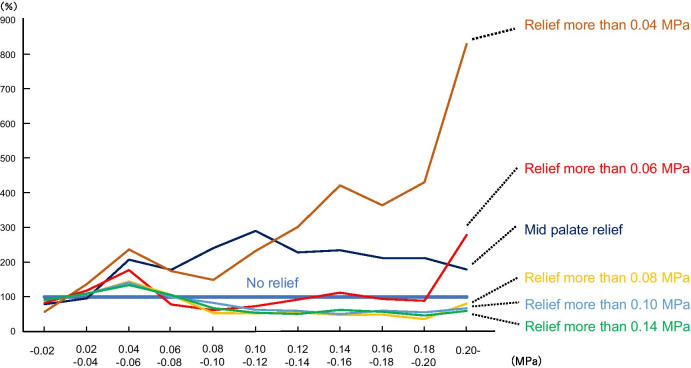
Comparison of relief model and no-relief model

Figure 6 shows the distribution volumes in the relief model relative to the situation when the distribution volume for each stress value in the no-relief model was considered to be 100%. In the relief model with a borderline of 0.04 MPa or higher, the distribution volume at which a high stress of 0.20 MPa or higher was generated was approximately 800% of that with the no-relief model, and in the relief model with a borderline of 0.06 MPa or higher, the respective ratio was approximately 280%. On the other hand, the relief models with borderline of 0.08 MPa or higher, 0.10 MPa or higher, and 0.14 MPa or higher were less than 100%, and among them, the relief models with values of 0.14 MPa or higher were approximately 60%. In the mid-palatal relief model, the distribution volume at which a stress of 0.20 MPa or higher was generated was 180% of that in the relief model.

## Discussion

### Subject and region of interest

The subject in this study was dentate, with healthy teeth and was selected as a reference to obtain useful data regarding the palatal mucosa of dentate patients before evaluating the denture-supporting mucosa of edentulous patients. Because this study covered only the mean palatal configuration of a single subject, it was confined to various aspects of the intraoral status of that subject, including the presence or absence of palatal tori, and palatal shape, with the primary focus being the development of analysis methods. In the future, it is necessary to investigate the differences due to palatal shape [9].

In addition, the simulated relief model is currently used in practice for creating pseudopalatal plates that reflect a relief based on each borderline in CAD/CAM that was output from the STL data. These plates were combined with a simultaneous measurement device [9] and were used to verify the simulated validity with subjects.

### Setting the borderline

In this study, the borderline for the simulation was set between 0.02, which was the level of stress generated in the palatal mucosa model, and 0.20 MPa, and the stress distribution was analyzed. It is difficult to set stress values for the onset of pain, but pain is thought to occur readily at relatively high stress values.

### Finite element analysis methods and analysis models

Analysis based on finite elements enables the partitioning of elements in the model, detailed setting of loading conditions and physical properties, and accurate simulation based on the introduction of temporal elements to the linear analysis and dynamic analysis. However, if analysis is performed with more detailed conditions set, considerable time is required. Therefore, for commercial use, in order to have a general appreciation of the entire tendency at the initial stages of structural design, sometimes only a first-order analysis is performed, that is, an approximate analysis, with the details simplified [14]. In the present study, the stress distribution was analyzed using an FEA model, and a simulation was performed for the stress distribution on the denture-supporting mucosa when relief was applied to ranges of the borderline and higher.

### Effects of relief on the palatal mucosa

In this study, the stress values increased by approximately 800% in the relief model with a borderline of 0.04 MPa or higher and by approximately 180% in the median relief model, compared with that in the no-relief model (Fig. 6). This suggests that excessive relief (e.g., relief model with a borderline of 0.04 MPa or higher) or standard relief (e.g., mid-palatal relief model) reduces the support area of the plates outside the relief range—this, in turn, causes excessive localized stress, which may lead to pain. On the other hand, in the relief model with a borderline of 0.14 MPa or higher, the high stress value was less than 100% compared with the no-relief model, and the stress was evenly distributed over the entire palatal mucosa. If relief can be applied locally, restricted to the sites of high stress generation, the supportive strength of the plate can probably be increased by making the stress consistent. In summary, the findings of this study suggest that relief applied based on the dentist’s subjective judgment and experience is not necessarily effective.

In this study, an FEA model was created using the elastic modulus of the palatal mucosa, mucosal thickness, and actual bite force measured in the oral cavity in previous studies [9], and the bite force and stress were analyzed in relation to the bite force at the time of pain generation; thus, it is necessary to consider strain in future studies. Additionally, we plan to validate the simulation by applying the structural optimization technology to change the stress value and the effect of repeated relief, and by using CAD/CAM to fabricate the pseudopalatal plate model created in this study and measuring the bite force during pain generation in an actual mouth.

## Conclusions

In this study, the high-stress distribution volume of the mid-palatal relief model increased in comparison with that of the no-relief model. On the other hand, in relief models with a borderline of 0.14 MPa or higher, stress was evenly distributed over the entire palatal mucosa. This suggests that the supportive strength of plates can be increased by selectively applying optimal relief rather than standard relief.

## Data Availability

Data may be made available from the corresponding author on reasonable request.

## Abbreviations

3D: Three-dimensional
FEA: Finite element analysis
